# The role of the nAChR subunits *α*5, *β*2, and *β*4 on synaptic transmission in the mouse superior cervical ganglion

**DOI:** 10.14814/phy2.14023

**Published:** 2019-03-19

**Authors:** Xenia Simeone, Rudolf Karch, Anna Ciuraszkiewicz, Avi Orr‐Urtreger, Rosa Lemmens‐Gruber, Petra Scholze, Sigismund Huck

**Affiliations:** ^1^ Division of Pathobiology of the Nervous System Center for Brain Research Medical University of Vienna Vienna Austria; ^2^ Institute of Biosimulation and Bioinformatics Center for Medical Statistics, Informatics, and Intelligent Systems Medical University of Vienna Vienna Austria; ^3^ Genetic Institute Tel Aviv Sourasky Medical Center and Sackler School of Medicine Tel Aviv University Tel Aviv Israel; ^4^ Department of Pharmacology and Toxicology University of Vienna Vienna Austria; ^5^ Present address: Research Group Molecular Physiology Leibniz Institute for Neurobiology Brenneckestraße 6 D‐39118 Magdeburg Germany

**Keywords:** Compound action potential, EPSP, Hexamethonium, knockout mice, nicotinic ACh receptor, superior cervical ganglion, synaptic transmission

## Abstract

Our previous immunoprecipitation analysis of nicotinic acetylcholine receptors (nAChRs) in the mouse superior cervical ganglion (SCG) revealed that approximately 55%, 24%, and 21% of receptors are comprised of *α*3*β*4, *α*3*β*4*α*5, and *α*3*β*4*β*2 subunits, respectively. Moreover, mice lacking *β*4 subunits do not express *α*5‐containing receptors but still express a small number of *α*3*β*2 receptors. Here, we investigated how synaptic transmission is affected in the SCG of *α*5*β*4‐KO and *α*5*β*2‐KO mice. Using an ex vivo SCG preparation, we stimulated the preganglionic cervical sympathetic trunk and measured compound action potentials (CAPs) in the postganglionic internal carotid nerve. We found that CAP amplitude was unaffected in *α*5*β*4‐KO and *α*5*β*2‐KO ganglia, whereas the stimulation threshold for eliciting CAPs was significantly higher in *α*5*β*4‐KO ganglia. Moreover, intracellular recordings in SCG neurons revealed no difference in EPSP amplitude. We also found that the ganglionic blocking agent hexamethonium was the most potent in *α*5*β*4‐KO ganglia (IC_50_: 22.1 *μ*mol/L), followed by *α*5*β*2‐KO (IC_50_: 126.7 *μ*mol/L) and WT ganglia (IC_50_: 389.2 *μ*mol/L). Based on these data, we estimated an IC_50_ of 568.6 *μ*mol/L for a receptor population consisting solely of *α*3*β*4*α*5 receptors; and we estimated that *α*3*β*4*α*5 receptors comprise 72% of nAChRs expressed in the mouse SCG. Similarly, by measuring the effects of hexamethonium on ACh‐induced currents in cultured SCG neurons, we found that *α*3*β*4*α*5 receptors comprise 63% of nAChRs. Thus, in contrast to our results obtained using immunoprecipitation, these data indicate that the majority of receptors at the cell surface of SCG neurons consist of *α*3*β*4*α*5.

## Introduction

Ganglia in the autonomic nervous system—particularly the sympathetic superior cervical ganglion (SCG)—have long been favored for studying neuronal nicotinic cholinergic synapses. The nicotinic acetylcholine receptors (nAChRs) expressed in the rodent SCG consist of homo‐pentameric *α*7 subunits, which bind *α*‐bungarotoxin (Brown and Fumagalli 1977), and hetero‐pentameric receptors containing *α*3, *β*4, *β*2, and/or *α*5 subunits (Mao et al. 2006; David et al. 2010). All hetero‐pentameric nAChRs in the SCG contain the “core” subunits *α*3 and *β*4, with *β*2 and *α*5 subunits serving as mutually exclusive accessory subunits (David et al. 2010) that confer distinct single‐channel properties to the receptor (Ciuraszkiewicz et al. 2013).

Mice that lack the *α*3 nAChR subunit, and double‐knockout mice lacking both the *β*2 and *β*4 subunits, display symptoms of autonomic deficiency and have impaired growth and increased perinatal mortality (Xu et al. 1999a,1999b). Disrupting the gene that encodes the *α*3 subunit eliminates both fast excitatory synaptic potentials in postganglionic sympathetic SCG neurons and eliminates ACh‐induced currents in SCG neurons cultured from 7‐day‐old mouse pups, thus establishing that *α*3‐containing postsynaptic nAChRs play a critical role in synaptic transmission (Rassadi et al. 2005). On the other hand, mice lacking the *β*4 subunit grow to adulthood and do not develop any visible phenotypic abnormalities, suggesting that *β*2 can replace *β*4 as a core receptor subunit (Xu et al. 1999b). Interestingly, however, deleting the *β*4 subunit not only dramatically reduces the total number of nAChRs measured using an immunoprecipitation assay (to <15% of control levels), but also eliminates all *α*5‐containing receptors (David et al. 2010). Mice lacking the *β*4 subunit have a reduced bradycardic response to high‐frequency (>40 pulses/s) vagal stimulation and are more sensitive to the ganglionic blocking compound hexamethonium (HM) (Wang et al. 2003). Similarly, mice lacking the *α*5 nAChR subunit also have an attenuated bradycardic response to high‐frequency vagal stimulation and are more sensitive to HM (Wang et al. 2002).

There is a more general interest in *α*5‐containing nAChRs co‐assembling with *α*3*β*4 or *α*4*β*2 in that these receptors play a key role in nicotine dependence (see e.g. Bierut et al. 2008; Forget et al. 2018). Genome‐wide association studies have indicated that single‐nucleotide polymorphisms (SNPs) within genes encoding nAChR subunits can substantially affect nAChR‐mediated smoking behavior in humans. Most prominent are the SNPs located within the CHRNA5/CHRNA3/CHRNB4 locus on chromosome 15q25, which encodes the *α*5, *α*3, and *β*4 nAChR subunits (see George et al. 2012). A non‐synonymous polymorphism (rs16969968), which changes the aspartic acid at position 398 in the *α*5 subunit to an asparagine (D398N), is strongly associated with a higher risk of increased nicotine consumption (Bierut et al. 2008).

It is therefore important to determine how the *α*5 subunit contributes to the function of *α*3*β*4 and/or of *α*4*β*2 nAChRs (see e.g. Tapia et al. 2007; Fowler et al. 2011; Frahm et al. 2011; Tammimaki et al. 2012; Chatterjee et al. 2013; Sciaccaluga et al. 2015; Deflorio et al. 2016). Here, we found that contrary to observations based on IP experiments (Mao et al. 2006; David et al. 2010), the majority of receptors at the cell surface of SCG neurons consist of *α*3*β*4*α*5, strengthening *α*3*β*4*α*5 receptor‐mediated cellular signaling.

## Methods

### Ethics approval

All experiments involving animals were performed in accordance with the European Communities Council directive (86/609/EEC) and Austrian federal law governing animal experimentation (Tierversuchsgesetz TVG 501/1989).

### Animals

Experiments were performed using either an intact SCG ex vivo preparation or in vitro cultured SCG neurons isolated from wild‐type C57BL/6J mice and mice carrying genetic deletions in the genes encoding the *α*5 (Wang et al. 2002), *α*5*β*4 (Kedmi et al. 2004), and *α*5*β*2 nAChR subunits. *α*5*β*2‐KO mice were generated by crossing *α*5‐KO and *β*2‐KO mice (generously provided by J.‐P. Changeux, Pasteur Institute, Paris, Picciotto et al. 1995). The mice used in this study were backcrossed onto the C57BL/6J background for six (*α*5*β*4), seven (*α*5), or 12 (*β*2) generations after germ line transmission. All animals were group‐housed in a climate‐controlled room at 21°C with a light:dark schedule of 10:14 h with free access to food and water.

### Cell culture of SCG neurons and ex vivo SCG preparation

For SCG neurons, mouse pups (3–5 days old) were killed by decapitation, and the SCGs were removed and treated with enzyme; dissociated neurons were plated on poly‐D‐lysine/laminin‐coated tissue culture dishes (Thermo Scientific‐Nunc) and cultured in 5% CO_2_ at 36.5°C with neurobasal medium supplemented with B27 (Gibco/Thermo Fisher Scientific) for 3–5 days prior to recording (Fischer et al. 2005).

**Figure 1:**
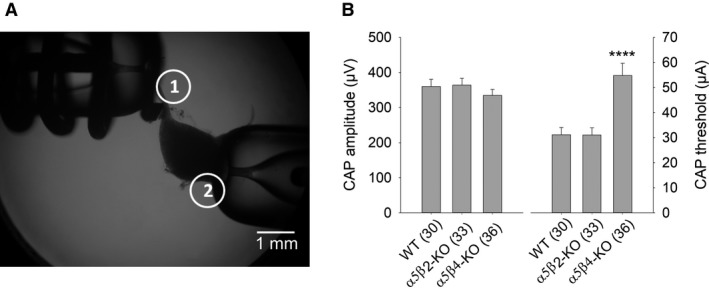
CAP amplitudes do not differ between WT,* α*5*β*2‐KO, and *α*5*β*4‐KO SCG ganglia. (A) Image showing the experimental setup for recording CAPs in the isolated mouse SCG. The suction electrode for stimulating the preganglionic sympathetic nerve is indicated as electrode 1, and the suction electrode for recording the postganglionic internal carotid nerve is indicated as electrode 2. (B) Summary of CAP amplitude (left) and the stimulus threshold for inducing a CAP (right) on WT,* α*5*β*2‐KO, and *α*5*β*4‐KO ganglia. CAP amplitude induced by supramaximal stimulation at 0.033 Hz was similar between genotypes (one‐way ANOVA,* F*_2,96_ = 0.75; *P *=* *0.48). In contrast, the stimulus threshold for eliciting a discernible CAP with an amplitude of 15–20 *μ*V was significantly higher in *α*5*β*4‐KO compared to both WT and *α*5*β*2‐KO mice (one‐way ANOVA, followed by Bonferroni's post hoc test, *P *<* *0.0001). In this and subsequent figures, summary data are presented as the mean ± SEM. See Figure 4 for example CAP recordings.

For ex vivo SCG experiments, adult mice (4–6 weeks old; 6–8 weeks old for intracellular recordings from *α*5*β*4‐KO mice) were anesthetized with CO_2_ and quickly decapitated with scissors. The head was immediately immersed in oxygenated Locke's solution (see below), pinned down to a Sylgard‐coated polystyrene preparation receptacle using four hypodermic needles, and carefully rinsed with Locke's solution until the liquid was completely clear. Using a stereo microscope, the innervating cervical sympathetic nerve was separated from the surrounding tissue. Next, the postganglionic internal carotid nerve was cut as far as possible from the body of the SCG. By grasping the connective tissue surrounding the SCG body and cutting the tissue behind the SCG, the ganglion was isolated from the neck and placed into a Petri dish filled with fresh oxygenated Locke's solution. For extracellular recordings of compound action potentials (CAPs), the ganglion was only roughly cleaned of excess fatty and connective tissue that would have floated around the superfused ganglion and possibly disturb the recording (see Fig. 1). For intracellular recordings of excitatory postsynaptic potentials (EPSPs), the protruding muscle and connective tissue were carefully removed from the ganglia, and the SCG was tightly pinned down on a Sylgard‐coated Petri dish using tungsten wire needles (25‐micron diameter).

### Solutions and reagents

Physiological Locke's solution containing (in mM) 136 NaCl, 20 NaHCO_3_, 8 glucose, 5.6 KCl, 1.2 NaH_2_PO_4_, 2.2 CaCl_2_•2H_2_O, and 1.2 MgCl_2_•6H_2_O was prepared as described previously (Briggs and McAfee 1988). The Locke's solution was bubbled with 95% O_2_/5% CO_2_ for at least 15–30 min before each experiment. At room temperature (21–23°C), the oxygenated Locke's solution had a pH value of 7.2.

The superfusion system consisted of silicone tubing (1 mm inner diameter) connected to two model 2120 Varioperpex II peristaltic pumps (LKB Instruments, Bromma, Sweden); one pump was used for the inflow of the superfusion solution and the other pump was used for the outflow. To minimize the electrical noise, a 2‐cm long stainless‐steel hypodermic needle connected to ground was intercalated in each tube. The superfusion flow rate was approximately 1 mL/min. All CAP and EPSP recordings were performed at room temperature. Where indicated, nicotine or hexamethonium (HM) was added to the oxygenated Locke's solution. During the experiments, all solutions were kept in 50‐mL centrifuge tubes and continuously bubbled with 95% O_2_/5% CO_2_. (−)‐Nicotine (N‐3876) and hexamethonium bromide (H‐0879) were purchased from Sigma‐Aldrich (St. Louis, MO), and all general chemical reagents were purchased from Merck‐VWR (Radnor, PA).

### Electrophysiological recordings

A Diaphot 300 phase‐contrast inverted microscope (Nikon) mounted on an anti‐vibration table was used to visualize the ganglia, nerves, and electrode tips. CAPs and EPSPs were induced by applying electrical pulses to the preganglionic cervical sympathetic trunk using a Master‐8 pulse stimulator (AMPI, Jerusalem, Israel) connected to an ISO‐Flex stimulus isolator (AMPI). CAPs were recorded from the postganglionic internal carotid nerve via a suction electrode connected to a differential electrometer (MetaMetrics AK47). Unless stated otherwise, we stimulated the preganglionic nerve using 50‐*μ*sec current pulses of variable amplitude delivered at 0.033 Hz. Starting with the smallest current amplitude, the stimulus intensity was slowly increased until the first discernible CAP appeared. To ensure that all possible fibers were activated, the experiments were performed at supramaximal intensity (i.e., the stimulus amplitude was increased to 110–115% of the stimulus amplitude that produced maximal CAPs). Prior to collecting data, the ganglia were recorded for 20 min to ensure that CAP amplitude was stable. Unstable CAPs and CAPs with diminishing amplitude indicated a loose fit between the nerve(s) and/or suction electrode(s); in such cases, the suction was re‐established, together with spare fat/connective tissue.

EPSPs were recorded from the ganglion body using a sharp intracellular electrode positioned with a 3‐axis stage micromanipulator (SM LN 1, Luigs & Neumann, Ratingen, Germany). The signal was pre‐amplified using a head stage (NPI, Tamm, Germany) and amplified using a single‐electrode amplifier (SEC 05 LX, NPI). The signals and the electrode potential were additionally monitored using two model HM 205‐3 storage oscilloscopes (HAMEG, Mainhausen, Germany). Data were digitized using a Digidata 1200 interface (Molecular Devices, Sunnyvale, CA) and stored on a personal computer via pClamp (Clampex, v.10.2, Molecular Devices). We recorded from neurons lying at the rostral part of the SCG, as this is the location of neurons that innervate the internal carotid nerve (Bowers and Zigmond 1979). Cells were considered suitable for use if the resting membrane potential (RMP) was stable at a value between −45 and −65 mV for 10 min; neurons with an RMP more positive than −45 mV, as well as neurons that had an overshoot that failed to reach 0 mV, were excluded. All recordings were considered stable if the baseline did not drift by more than 3%. EPSPs were induced using 50‐*μ*sec current pulses of variable amplitude delivered at 0.33 Hz. The stimulus was set to 100 msec after the start of the recording. To monitor membrane resistance, a 20‐msec depolarizing current pulse was applied after the action potential (AP) or EPSP returned to RMP (i.e., 350 msec from the start of the recording sweep).

Patch‐clamp recordings of cultured SCG neurons were performed at room temperature using the perforated‐patch technique (Rae et al. 1991). The recording equipment, superfusion system, and bath and pipette solutions for these experiments have been described previously (Fischer et al. 2005).

### Data analysis

All data were recorded using Clampex 10.2 and analyzed with Clampfit 10.2 (pClamp 10, Molecular Devices). To measure CAP amplitude, 30 traces were averaged and low‐pass filtered at 2 kHz (Gaussian). CAP amplitude was measured between one cursor set just before the onset of the stimulus artifact and a second cursor set at the maximum value.

To measure EPSP amplitude, the membrane was hyperpolarized to −100 mV, and 20 traces were averaged and low‐pass filtered at 2 kHz (Gaussian). EPSP amplitude was measured between one cursor set just before the onset of the stimulus artifact and a second cursor set at the maximum value. The slope of the EPSP decay was measured between a cursor set 4 msec after the maximum value (corresponding to approximately 90% of the amplitude) and a cursor set approximately 350 msec from the start of the sweep (i.e., just before the onset of the depolarizing step used to monitor membrane resistance). The decay phase was fit to a double‐exponential equation using the least‐squares Levenberg Marquardt algorithm as follows:

(1) $$f\left(t\right)=\sum_{i=1}^{n}A_{i}e^{-t/\tau_{i}}+C$$

where *n* is 2, *A* is the amplitude, and *τ* the time constant, respectively, for each component *i*, and *C* is the constant *y*‐offset.

### Statistics

Statistics and curve‐fitting analysis were performed using GraphPad Prism (v7.04, GraphPad Software, La Jolla, CA). Data were then exported into SigmaPlot (v13, Systat Software Inc., San Jose, CA) in order to draw the plots. All data were assessed for normality using the D'Agostino & Pearson test and thereafter analyzed using either an ANOVA or Student's *t*‐test. CAP amplitude, CAP threshold, and EPSP amplitude and decay were analyzed using a one‐way independent measures ANOVA followed by Bonferroni's multiple comparison in order to assess the effect of genotype and/or treatment. For the CAP experiments with increasing pulse frequency, the data were analyzed using a two‐way repeated measures ANOVA (with pulse number as the repeated factor) followed by Bonferroni's multiple comparison. The data points in the concentration‐response curves were fitted using nonweighted nonlinear regression. An *F*‐test was used to test for different versus shared (identical) IC_50_ values. Differences are considered significant at *P *≤* *0.05. In the figures, *P ≤* *0.05, **P ≤* *0.01, ***P ≤* *0.001, and ****P ≤* *0.0001. Details regarding our algorithm for estimating the IC_50_ of HM on *α*3*β*4*α*5 receptors, and the relative contribution of these receptors to the total number of hetero‐pentameric nAChRs in the SCG, are provided in the Results section.

## Results

### CAPs in *α*5*β*4‐KO ganglia differ from CAPs in WT and *α*5*β*2‐KO ganglia with respect to the activation threshold but not amplitude

Here, we included wild‐type (WT) mice and mice lacking *α*5*β*4 (*α*5*β*4‐KO) and *α*5*β*2 (*α*5*β*2‐KO) receptors (expressing *α*3*β*2 and *α*3*β*4 hetero‐pentameric receptors, respectively). We also studied mice lacking *α*7 subunits, but because we found no difference between these mice and WT mice with respect to CAP recordings (data not shown), the data obtained with *α*7‐KO mice are not discussed further (see also Brown and Fumagalli 1977).

First, we addressed the question of how synaptic transmission is maintained in mice lacking the *β*4 subunit, as all hetero‐pentameric receptors in the SCG contain this subunit. Using immunoprecipitation (IP), we previously reported that loss of the *β*4 subunit also eliminates *α*5‐containing receptors and reduces the number of nAChRs by >85 (David et al. 2010). Nevertheless, for this study, we used SCGs from *α*5*β*4 double‐KO mice, which express exclusively *α*3*β*2 hetero‐pentameric nAChRs.

Interestingly, supramaximal stimulation of the afferent cervical sympathetic trunk in *α*5*β*4‐KO SCG induced CAPs with an amplitude that did not differ significantly from either WT or *α*5*β*2‐KO SCG (Fig. 1). Thus, because the CAP represents the sum of all APs in the postganglionic nerve, postganglionic neurons are activated to the same extent in *α*5*β*4‐KO and WT ganglia, despite a significant reduction in nAChRs in *α*5*β*4‐KO ganglia. Interestingly, however, the threshold required to elicit a CAP in *α*5*β*4‐KO ganglia was higher than in both *α*5*β*2‐KO and WT ganglia (Fig. 1).

### Varying stimulation frequency reveals differences in pulse‐dependent CAP amplitude between WT and KO SCG

**Figure 2:**
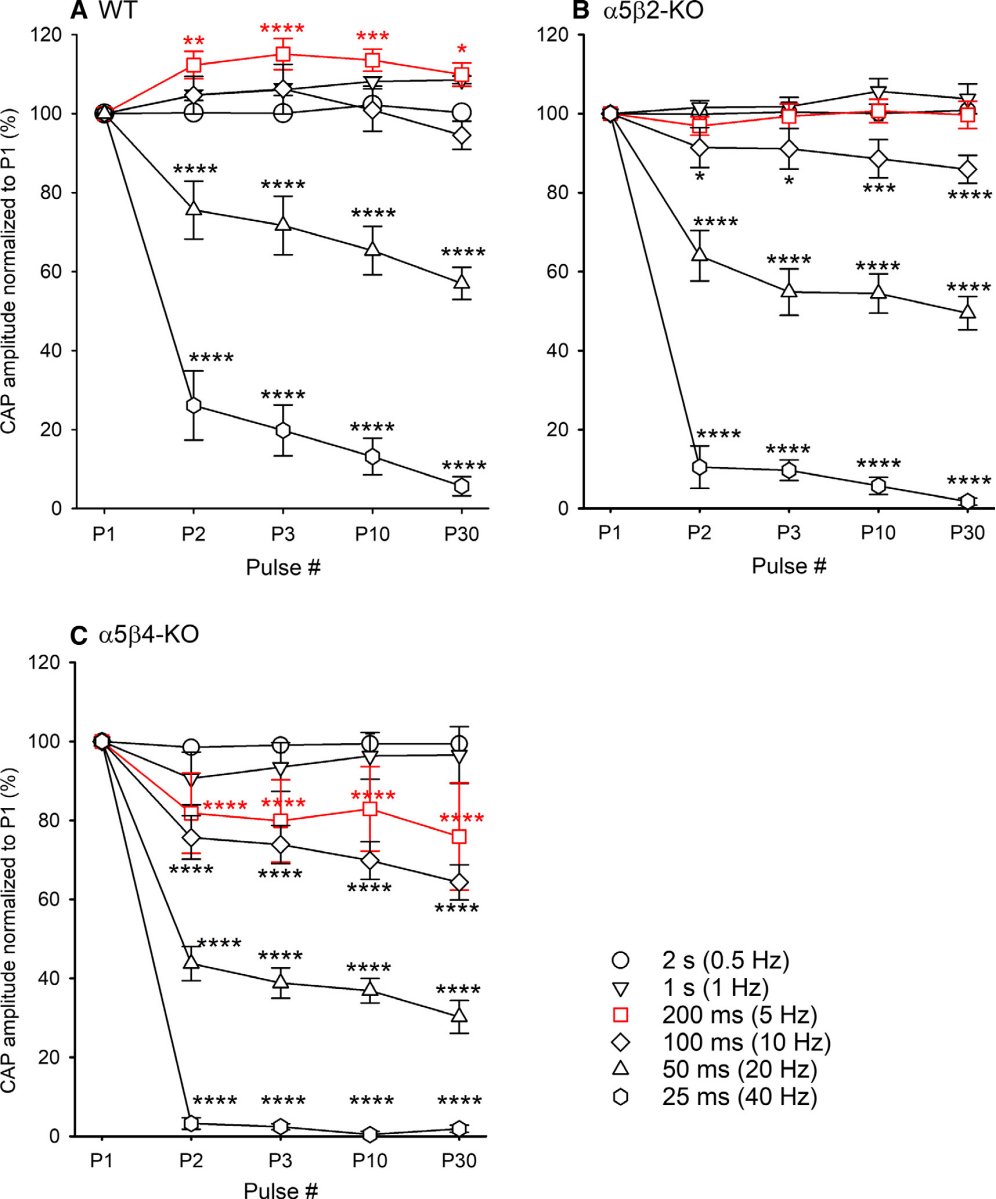
Synaptic transmission is more susceptible to increasing stimulation frequency in *α*5*β*2‐KO and *α*5*β*4‐KO ganglia than in WT ganglia. CAP amplitude was measured in WT (A), *α*5*β*2‐KO (B), and *α*5*β*4‐KO (C) ganglia in response to a train of 30 supramaximal pulses delivered at increasing frequency and normalized to the amplitude of the first CAP (*n* = 7 ganglia per genotype). For clarity, only the first, second, third, 10th, and 30th pulses are shown. Two‐way repeated ANOVA using the frequency as the group factor and the pulse numbers as repeated factor showed highly significant pulse effects for (A) WT ganglia (*F*_4, 144_ = 60.76; *P *<* *0.0001), (B) *α*5*β*2‐KO ganglia (*F*_4, 144_ = 163.6; *P *<* *0.0001), and (C) *α*5*β*4‐KO ganglia (*F*_4, 144_ = 204.1; *P *<* *0.0001). Significant differences to the first pulse were calculated by Bonferroni's post hoc test.

Using our standard stimulation protocol of 0.033 Hz (i.e., delivering pulses at 30‐sec intervals), we produced stable responses with respect to CAP amplitude in all three genotypes (see Fig. 4A1 for representative examples). We therefore examined whether increasing stimulation frequency could reveal any differences between WT and KO ganglia. To address this question, we stimulated the SCG using six frequencies (0.5, 1, 5, 10, 20, and 40 Hz) and measured normalized CAP amplitude elicited by the first, second, third, 10th, and 30th pulses (Fig. 2). We found that stimuli delivered at 0.5 Hz and 1 Hz had no significant effect on CAP amplitude in all three genotypes. Interestingly, however, stimuli delivered at 5 Hz (i.e., at 200‐msec intervals) caused a significant increase in CAP amplitude in WT ganglia, had no effect in *α*5*β*2‐KO ganglia, and significantly decreased CAP amplitude in *α*5*β*4‐KO ganglia. Applying stimuli at the highest frequencies (20 Hz and 40 Hz) significantly decreased CAP amplitude in all three genotypes, with the strongest effect in *α*5*β*4‐KO ganglia (Fig. 2C).

Next, we focused on the first two pulses delivered at 5 Hz (i.e., we applied a paired‐pulse paradigm with a 200‐msec interval between pulses). We then re‐analyzed the data in order to compare the difference in CAP amplitude between the first and second pulses using a paired Student's *t*‐test. Our analysis revealed significant paired‐pulse facilitation in WT ganglia (P2/P1 = 1.12 ± 0.03; *P *=* *0.0094, *n* = 7); in contrast, no paired‐pulse facilitation or depression was observed in *α*5*β*2‐KO ganglia (P2/P1 = 0.97 ± 0.02; *P *=* *0.31, *n* = 7), whereas significant paired‐pulse depression was observed in *α*5*β*4‐KO ganglia (P2/P1 = 0.84 ± 0.08; *P *=* *0.04, *n* = 7).

### The potency of nicotine at inhibiting CAP amplitude differs between *α*5*β*4‐KO, *α*5*β*2‐KO, and WT ganglia

**Figure 3:**
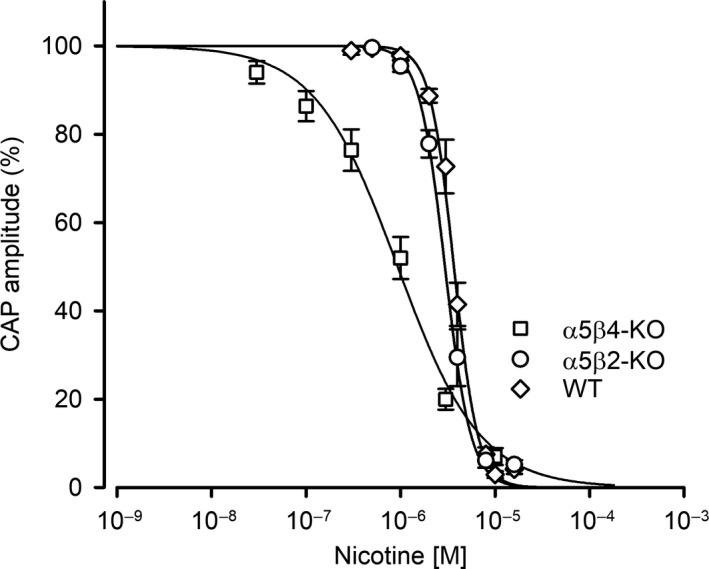
The potency of nicotine at inhibiting CAP amplitude differs between *α*5*β*4‐KO,* α*5*β*2‐KO, and WT ganglia. Nicotine is more potent at inhibiting CAP amplitude in *α*5*β*4‐KO ganglia (squares) compared to both WT (circles) and *α*5*β*2‐KO (up‐triangles) ganglia. Nicotine concentration‐response curves show that CAPs following supramaximal stimulation at 0.033 Hz were most potently reduced in *α*5*β*4‐KO (IC_50_ = 0.93 *μ*mol/L, confidence interval: 0.78–1.11 *μ*mol/L, *n* = 15), followed by *α*5*β*2‐KO (3.01 *μ*mol/L, confidence interval: 2.80–3.23 *μ*mol/L, *n* = 14) and WT ganglia (3.67 *μ*mol/L, confidence interval: 3.50–3.85 *μ*mol/L, *n* = 13). The IC_50_ values for *α*5*β*4‐KO and *α*5*β*2‐KO (*F*_1,145_ = 69.1, *P *<* *0.0001, *F*‐test), and for *α*5*β*2‐KO and WT differ significantly (*F*_1,139_ = 21.0, *P = <*0.0001, *F*‐test).

Next, we examined the effect of increasing concentrations of nicotine at reducing CAP amplitude. Our analysis revealed that nicotine decreased CAP amplitude in a concentration‐dependent manner, consistent with desensitization of the nAChRs expressed in SCG neurons (Fig. 3). Calculating the IC_50_ values revealed that nicotine was more potent in *α*5*β*4‐KO ganglia (i.e., ganglia expressing *α*3*β*2 receptors) compared to both *α*5*β*2‐KO and WT ganglia, with IC_50_ values of 0.93, 3.01, and 3.67 *μ*mol/L, respectively. Thus, the IC_50_ differed significantly between *α*5*β*2‐KO and *α*5*β*4‐KO ganglia (*F*_1,145_ = 137.3, *P *<* *0.0001, *F*‐test) and between *α*5*β*2‐KO and WT ganglia (*F*_1,139_ = 21.0, *P *<* *0.0001, *F*‐test).

### The difference in hexamethonium (HM) potency allows conclusions regarding *α*5‐containing receptors at the neuronal cell surface

**Figure 4:**
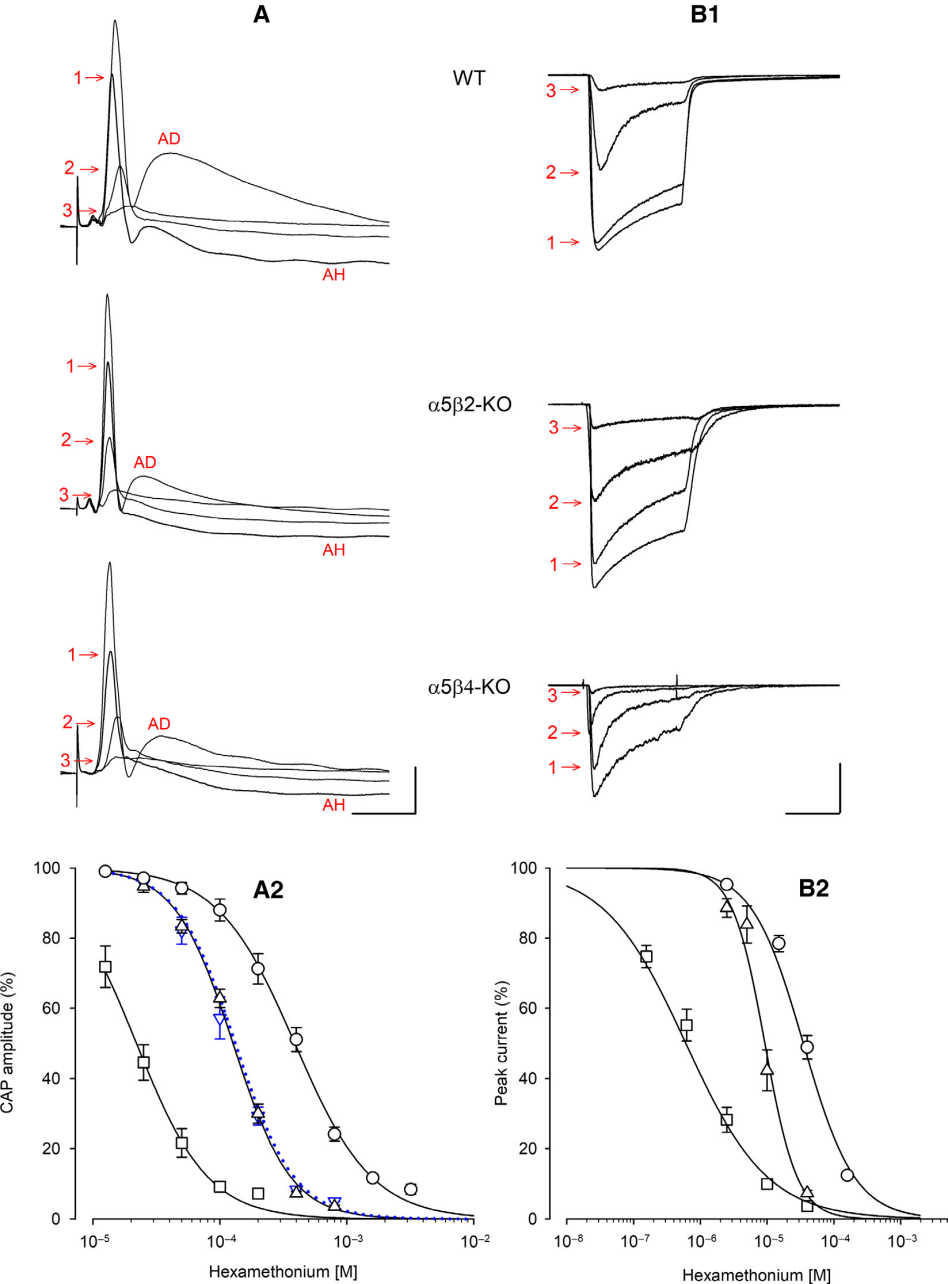
Hexamethonium (HM) inhibits CAP amplitude (in SCG ganglia) and ACh‐induced current (in cultured neurons) differently between WT,* α*5*β*2‐KO, and *α*5*β*4‐KO. Example traces and HM concentration‐response curves measured in WT (circles), *α*5*β*2‐KO (up‐triangles), and *α*5*β*4‐KO (squares) ganglia/neurons. (A1) Example traces of CAPs recorded in the absence or presence of HM. HM concentrations were 200 *μ*mol/L (1), 800 *μ*mol/L (2), and 1600 *μ*mol/L (3) in WT ganglia; 100 *μ*mol/L (1), 200 *μ*mol/L (2), and 400 *μ*mol/L (3) in *α*5*β*2‐KO ganglia; and 25 *μ*mol/L (1), 50 *μ*mol/L (2), and 100 *μ*mol/L (3) in *α*5*β*4‐KO ganglia. Afterdepolarization (AD, prominent in the absence of HM) and afterhyperpolarization (AH, prominent in the presence of low HM concentrations) are likely due to Ca^2+^‐dependent activation of Cl^−^ and K^+^ channels, respectively (Martinez‐Pinna et al. 2000). The horizontal and vertical scale bars represent 20 msec and 100 *μ*V, respectively. (A2) HM concentration‐response curves of CAP inhibition. CAPs as shown in panel A1 were most potently inhibited in *α*5*β*4‐KO ganglia (IC_50_ = 22.1 *μ*mol/L, confidence interval: 18.7–25.3 *μ*mol/L; Hill coefficient: −1.54; *n* = 10), followed by *α*5*β*2‐KO ganglia (126.7 *μ*mol/L, confidence interval: 120.2–133.5 *μ*mol/L; Hill coefficient: −1.9; *n* = 11) and WT ganglia (389.2 *μ*mol/L, confidence interval: 356.0–425.5 *μ*mol/L; Hill coefficient: −1.43; *n* = 10). The IC_50_ values for WT and *α*5*β*2‐KO ganglia differed significantly (*F*_1,111_ = 424.5, *P *<* *0.0001, *F*‐test). The IC_50_ of *α*5 single KO ganglia (119.0 *μ*mol/L, confidence interval: 109.3–129.6 *μ*mol/L, *n* = 6; down‐triangles, blue dotted line) did not differ from the IC_50_ of *α*5*β*2‐KO ganglia (*F*_1,113_ = 1.716, *P *=* *0.193, *F*‐test). (B1) Example traces of currents induced by 2 sec pulses of 300 *μ*mol/L ACh in the absence or presence of HM. HM concentrations were 10 *μ*mol/L (1), 40 *μ*mol/L (2), and 160 *μ*mol/L (3) in WT neurons; 2.5 *μ*mol/L (1), 10 *μ*mol/L (2), and 40 *μ*mol/L (3) in *α*5*β*2‐KO neurons; and 0.625 *μ*mol/L (1), 2.5 *μ*mol/L (2), and 10 *μ*mol/L (3) in *α*5*β*4‐KO neurons. The horizontal scale bar represents 1 s. The vertical scale bar represents 2 nA and 1 nA for *α*5*β*2‐KO and *α*5*β*4‐KO neurons, respectively. (B2) HM concentration‐response curves of peak current inhibition. Currents as shown in panel B1 were most potently inhibited in *α*5*β*4‐KO neurons (IC_50_ = 0.73 *μ*mol/L, confidence interval: 0.59–0.89 *μ*mol/L, Hill coefficient: −0.77; *n* = 17 neurons), followed by *α*5*β*2‐KO (9.28 *μ*mol/L, confidence interval: 8.07–10.79 *μ*mol/L: Hill coefficient: −1.8; *n* = 8) and WT (35.08 *μ*mol/L, confidence interval: 30.61–40.15 *μ*mol/L; Hill coefficient: −1.13; *n* = 20). The IC_50_ values in *α*5*β*2‐KO and WT differ significantly (*F*_1,86_ = 146, *P *<* *0.0001, *F*‐test).

Wang et al. (2002) previously reported that compared to WT mice, mice lacking the *α*5 nAChR subunit were more sensitive to the classic nAChR antagonist hexamethonium (HM) with respect to preventing bradycardia induced by high‐frequency stimulation of the vagus nerve. Our data derived directly from measuring synaptic transmission confirm these observations and provide further insight into the properties of nAChRs in the SCG. By applying suprathreshold stimuli to the preganglionic nerve, we recorded CAPs that were followed by prominent afterdepolarization (Fig. 4A1). Relatively low concentrations of HM inhibited this afterdepolarization and revealed an additional hyperpolarizing component, which was inhibited by high concentrations of HM (Fig. 4A1). The depolarization and the hyperpolarization are likely due to Ca^2+^‐activated Cl^−^ channels and Ca^2+^‐activated K^+^ channels, respectively (Martinez‐Pinna et al. 2000). We found that HM is significantly more potent at inhibiting CAPs in *α*5‐KO ganglia (IC_50_: 119.0 *μ*mol/L) compared to WT ganglia (IC_50_: 389.2 *μ*mol/L) (Fig. 4A2). The additional deletion of the *β*2 subunit (i.e., *α*5*β*2‐KO) had no further effect on the potency of HM (IC_50_: 126.7 *μ*mol/L), indicating that *α*3*β*4*β*2 receptors do not contribute to the effects of HM and are—at least in this respect—similar to *α*3*β*4 receptors. In contrast, deleting the *β*4 subunit (i.e., *α*5*β*4‐KO) caused a significant left‐shift in the dose‐response curve, resulting in significantly higher potency (IC_50_: 22.1 *μ*mol/L) compared to *α*5‐KO ganglia (Fig. 4A2).

Using IP, we previously showed that all hetero‐pentameric nAChRs in the SCG of WT animals contain *α*3 and *β*4 subunits, and that 21% and 24% of nAChRs also contain the *β*2 or *α*5 subunit, respectively; moreover, we found that the *α*5 and *β*2 subunits are mutually exclusive and are never present in the same receptor (David et al. 2010). The difference in the potency of HM between WT ganglia and *α*5‐KO (and *α*5*β*2‐KO) ganglia enabled us to estimate how many *α*5‐containing receptors in the WT SCG are required for the observed right‐shift in the HM concentration‐response curve for *α*3*β*4 receptors.

To model the concentration‐response curve of HM‐induced CAP inhibition for a combination of *α*3*β*4 and *α*3*β*4*α*5 receptors (i.e., in the WT SCG), we applied a weighted sum of two Hill functions as follows:

(2) $$y=f_{1}\cdot \frac{y_{\max}\cdot x^{n}}{\text{IC}50_{a}^{n}+x^{n}}+\left(1-f_{1}\right)\cdot \frac{y_{\max}\cdot x^{n}}{\text{IC}50_{b}^{n}+x^{n}}$$

where *x* is the concentration of HM, *y* is the CAP amplitude (expressed as a percent), *f*_1_ is the fraction of the effect contributed by *α*3*β*4 receptors, (1‐*f*_1_) is the fraction of the effect contributed by *α*3*β*4*α*5 receptors, *n* is the Hill coefficient, and *y*_max_ is normalized to the maximum CAP amplitude measured in the absence of HM (i.e., 100%). IC_50a_ and IC_50b_ are the HM concentrations that produce a 50% inhibition of *α*3*β*4 receptors (data derived from the *α*5*β*2‐KO) and *α*3*β*4*α*5 receptors (determined as a fit parameter), respectively. The above model was fitted to the experimental data obtained for WT mice using the *nlinfit* function of the *Statistics and Machine Learning Toolbox* of MATLAB (R2018a, MathWorks, Natick, MA, USA). The fit parameters were *f*_1_ and the IC_50b_, whereas the Hill coefficient *n* was taken from *α*5*β*2‐KO and is assumed to be the same for both *α*3*β*4 (the first term) and *α*3*β*4*α*5 (the second term). Goodness‐of‐fit was assessed by visual inspection of the observed (in WT) and predicted (by the model) responses and by the asymptotic standard errors of parameter estimates.

**Figure 5:**
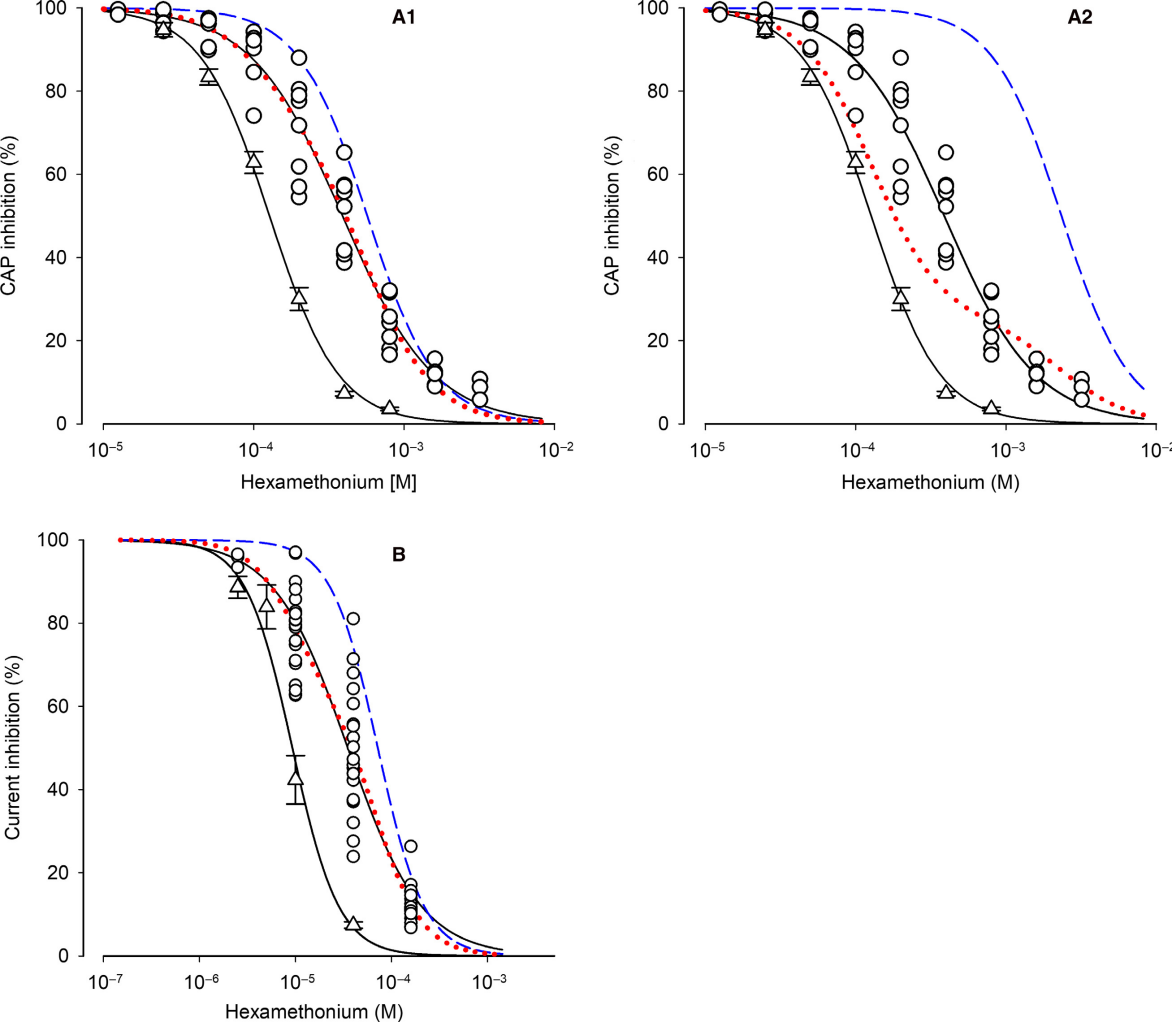
Modeling of the contribution of *α*5‐containing receptors in synaptic transmission and ACh‐induced currents in WT SCG. (A1) HM concentration‐response curves of CAP inhibition for *α*5*β*2‐KO and WT ganglia are taken from Fig. 4A2. Circles are the individual data points observed in WT ganglia. The blue dashed curve was generated for the HM inhibition of *α*3*β*4*α*5 receptors by applying a Hill coefficient −1.9 and the fit parameter IC_50b_ = 568.6 *μ*mol/L derived from Function (2) to the Hill function. The red dotted curve was generated by applying the following values to Function (2): *f*1 = 0.28 (the contribution of receptors lacking *α*5, derived from the fitting routine), (1‐f1) = 0.72 (the complementary contribution of *α*3*β*4*α*5 receptors), IC_50a_ = 126.7 *μ*mol/L (IC_50_ for *α*3*β*4 receptors), IC_50b_ = 568.6 *μ*mol/L (IC_50_ for *α*3*β*4*α*5 receptors, derived from the fitting routine), and the Hill coefficient −1.9. Please note the almost perfect overlay of the red dotted curve and the HM concentration‐response curve in WT ganglia. Based on this modeling approach, *α*3*β*4*α*5 receptors contribute 72% to overall nAChRs that mediate synaptic transmission in the mouse SCG. Details of the fitting routine: IC_50b_ = 568.6 *μ*mol/L ± 58.24 *μ*mol/L (10.2%); *f*_1_ = 0.28 ± 0.05 (19%); final sum of squares of residuals: 2498.46; degrees of freedom: 48; rms of residuals: 7.21466; variance of residuals: 52.0514. (A2) The plot differs from the plot shown in A1 by the blue dashed and the red dotted curves. Here, the fitting routine was constrained by inserting a fixed f_1_ of 0.75 into Function (2), which reflects the proportion of receptors lacking the *α*5 subunit (as determined by previous IP experiments, David et al. 2010). Even with this constraint, the fit converged by calculating a HM IC_50b_ of 2.35 mM (for *α*3*β*4*α*5 receptors, blue dashed curve). The red dotted curve was thereafter generated by applying the following values to Function (2): f1 = 0.75, (1‐f1) = 0.25, IC_50a_ = 126.7 *μ*mol/L, IC_50b_ = 2.35 mM, and the Hill coefficient −1.9. Please note that the red dotted curve clearly deviates from the data observed in WT ganglia. Details of the fitting routine: IC_50b_ = 2.35 mM ± 1.11 *μ*mol/L (47.3%); f_1_ = fixed (0.75); final sum of squares of residuals: 12082; degrees of freedom: 49; rms of residuals: 15.70; variance of residuals: 246.57. Besides the (calculated) high IC_50b_ for HM, please also note its high variability. (B) HM concentration‐response curves of peak current inhibition in *α*5*β*2‐KO and WT neurons are taken from Fig. 4B2. Circles are the individual data points observed in WT SCG neurons. The blue dashed curve was generated for the HM inhibition of *α*3*β*4*α*5 receptors by applying a Hill coefficient ‐1.8 and the fit parameter IC_50b_ = 72.02 *μ*mol/L derived from Function (2). The red dotted curve was generated by applying the following values to Function (2): f1 = 0.37 (the contribution of receptors lacking *α*5, derived from the fitting routine), (1‐f1) = 0.63 (the complementary contribution of *α*3*β*4*α*5 receptors), IC_50a_ = 9.28 *μ*mol/L (IC_50_ for *α*3*β*4 receptors), IC_50b_ = 72.02 *μ*mol/L (IC_50_ for *α*3*β*4*α*5 receptors, derived from the fitting routine), and the Hill coefficient −1.8. Please note the almost perfect overlay of the red dotted curve and the HM concentration‐response curve in WT ganglia. Based on this modeling approach, *α*3*β*4*α*5 receptors contribute 63% to overall nAChRs that mediate synaptic transmission in the mouse SCG. Details of the fitting routine: IC_50b_ = 72.02 *μ*mol/L ± 10.57 *μ*mol/L (14.7%); f_1_ = 0.37 ± 0.04 (12.5%); final sum of squares of residuals: 6641; degrees of freedom: 59; rms of residuals: 10.61; variance of residuals: 112.0.

Applying the IC_50a_ for *α*3*β*4 receptors (126.7 *μ*mol/L) and a fixed Hill coefficient (*n*) of −1.9 yielded a fit that converged with the estimated values of *f*_1_ = 0.28 and an IC_50b_ = 568.6 *μ*mol/L (see Fig. 5A1 for details). Thus, the potency of HM differs between *α*3*β*4 (119.0 *μ*mol/L) and *α*3*β*4*α*5 receptors (568.6 *μ*mol/L) by a factor of approximately 5. More importantly, our modeling results suggest that *α*3*β*4*α*5 receptors outnumber *α*3*β*4 receptors at synapses (72% vs. 28%, respectively), which is the opposite of what our IP experiments showed (David et al. 2010). When assigning 0.75 as a fixed value for *f*_1_ (i.e., 75% receptors lacking *α*5, based on our IP observations), the fit did converge, but with an HM IC_50b_ of 2.35 mmol/L for *α*3*β*4*α*5 receptors. Moreover, the fitted curve clearly deviated from the experimental data obtained in WT mice (Fig. 5A2).

Based on these results, our modeling approach suggests that the percentage of *α*5‐containing receptors at synaptic sites in the SCG is considerably higher than was predicted based on analyzing solubilized receptors. We therefore investigated whether this observation applies only to synaptic sites, or whether the *α*5‐containing receptors on the cell surface are also expressed at a higher percentage than was predicted by our IP experiments. To address this question, we cultured SCG neurons for 3–5 days, measured the peak currents induced by 2‐sec pulses of 300 *μ*mol/L ACh (in the presence of 0.1 *μ*mol/L atropine), and determined the effects of applying increasing concentrations of HM (Fig. 4B).

Similar to our ex vivo results obtained with intact ganglia, our in vitro experiments revealed that HM was most potent in *α*5*β*4‐KO neurons (IC_50_ = 0.73 *μ*mol/L), followed by *α*5*β*2‐KO neurons (9.28 *μ*mol/L) and WT (35.08 *μ*mol/L) neurons (Fig. 4B2). Thus, HM is approximately four times more potent in *α*5*β*2‐KO neurons compared to WT neurons, which is similar to the fivefold difference measured in intact ganglia.

Applying the abovementioned modeling approach to the in vitro data, the fit converged with a fixed Hill coefficient (*n*) of −1.8 (similar to the *α*3*β*4 receptors measured in *α*5*β*2‐KO ganglia), an *f*_1_ value of 0.37, and an IC_50b_ of 72.02 *μ*mol/L (see Fig. 5B for details). Thus, the potency of HM differed between *α*3*β*4 (9.28 *μ*mol/L) and *α*3*β*4*α*5 receptors (72.02 *μ*mol/L) by a factor of approximately 8. Moreover, and similar to our results based on synaptic receptors, our modeling approach suggests that *α*3*β*4*α*5 receptors also outnumber receptors lacking *α*5 (*α*3*β*4 and *α*3*β*4*β*2) at the cell surface in general (63% vs. 37%, respectively). Taken together, the two distinct preparations (CAP measurements using an ex vivo system and whole‐cell recordings in cultured neurons) yielded similar results, indicating that approximately 63–72% of functional nAChRs in the WT SCG contain the *α*5 subunit.

### EPSP amplitude is not altered in SCG lacking specific nAChR subunits

Next, we measured synaptic transmission at the single‐cell level in our three genotypes by recording excitatory postsynaptic potentials (EPSPs). To elicit a postsynaptic response, we applied 50‐*μ*sec pulses of approximately 15–20 *μ*A to the preganglionic nerve (the mean current amplitude required to induce an EPSP in WT, *α*5*β*2‐KO, and *α*5*β*4‐KO ganglia was 20.6 ± 8.5, 15.6 ± 2.7, and 18.6 ± 8.2 *μ*A, respectively), which is approximately half the current required to elicit a discernible CAP (see Fig. 1). Unlike our CAP recordings, we found no significant difference in stimulation thresholds between genotypes. Similarly, we found that RMP was similar between WT, *α*5*β*2‐KO, and *α*5*β*4‐KO neurons, with mean RMP values of −49.3 ± 2.3, −48.5 ± 2.3, and −53.3 ± 2.9 mV, respectively). When tested at their RMP, all responding cells produced an action potential (AP) at the threshold stimulus amplitude, which indicates that either all inputs triggered by the minimal stimulus were “strong”, or that multiple EPSPs (resulting from “weak” inputs) were activated simultaneously (Wang et al. 2010). Nevertheless, we found that the amplitude of APs was similar between WT, *α*5*β*2‐KO, and *α*5*β*4‐KO neurons (64.5 ± 4.3, 65.8 ± 3.2, and 63.7 ± 3.3 mV, respectively).

**Figure 6:**
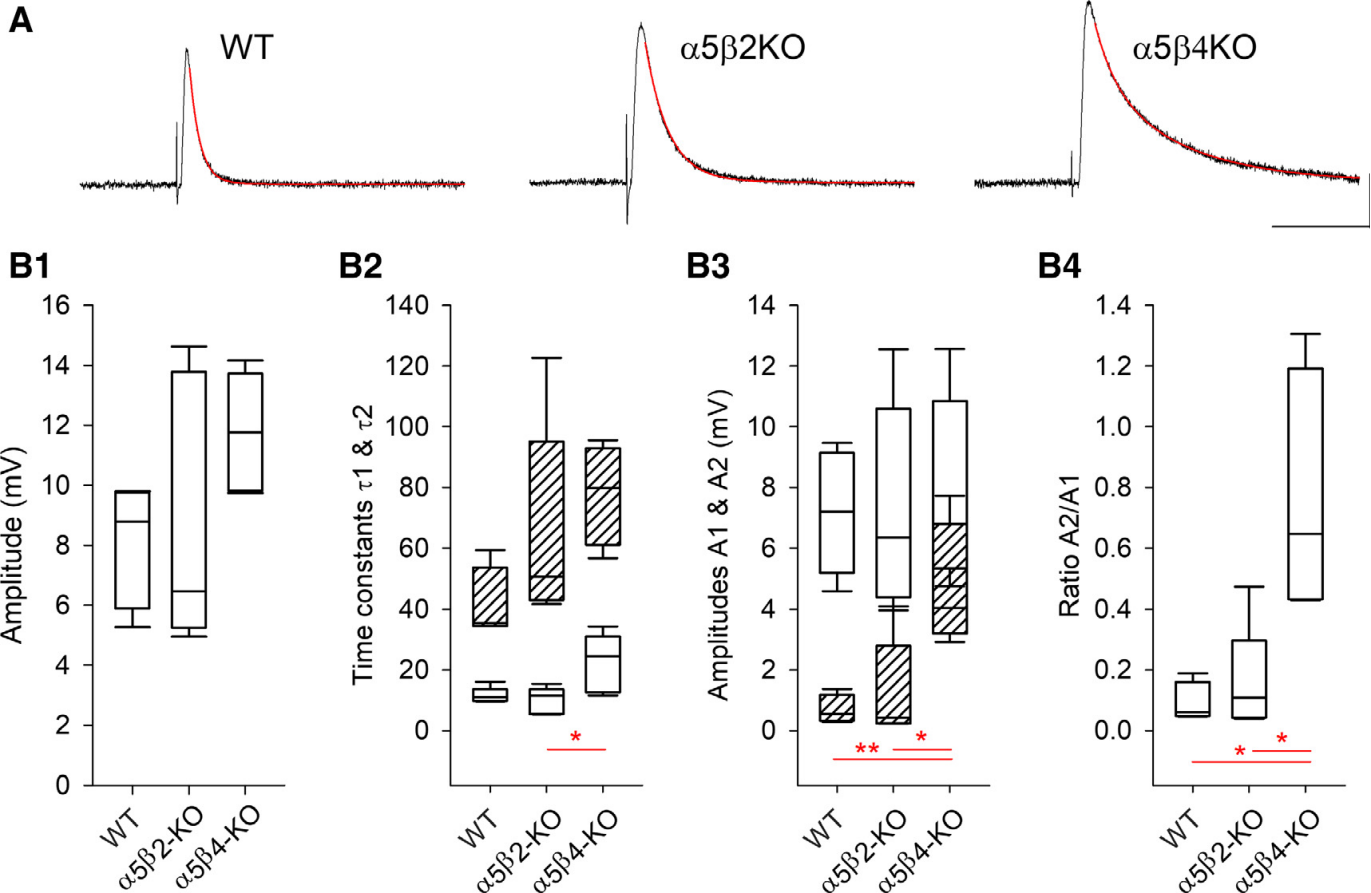
EPSP amplitudes do not differ between WT,* α*5*β*2‐KO, and *α*5*β*4‐KO SCG neurons. (A) Example EPSPs recorded in WT, *α*5*β*2‐KO, and *α*5*β*4‐KO ganglia; the decay phase was fit to a double‐exponential function (red lines). The cells were hyperpolarized to −100 mV with current injection, and EPSPs were induced by stimulating the preganglionic nerve. The horizontal and vertical scale bars represent 100 msec and 4 mV, respectively. (B) Box plots summarizing amplitudes (B1) and the fitting parameters for the decay phase of the EPSPs recorded in WT, *α*5*β*2‐KO, and *α*5*β*4‐KO neurons (*n* = 5 per genotype). In panel B2, the open and hatched boxes represent the fast and slow time constants, respectively; in panel B3, the open and hatched boxes represent the amplitudes corresponding to the fast and slow time constants, respectively.

Next, we measured both the amplitude and time course of EPSPs by hyperpolarizing the cells from their RMP to −100 mV, thus increasing the cell's threshold for generating an AP. Under these conditions, the EPSP amplitude in WT, *α*5*β*2‐KO, and *α*5*β*4‐KO neurons was 8.00 ± 0.91, 8.90 ± 2.03, and 11.77 ± 0.89 mV, respectively (Fig. 6). We also fit the declining phase of the EPSPs using a double‐exponential function (Fig. 6B). Although the time constant for the slow component was similar between all three genotypes (*F*_2, 10_ = 2.43, *P *=* *0.1382), the fast component significantly differed between *α*5*β*4‐KO and *α*5*β*2‐KO neurons (one‐way ANOVA: *F*_2, 12_ = 5.714, *P *=* *0.0181; Bonferroni's post hoc multiple comparisons test: *P *=* *0.0269). Interestingly, however, despite this moderate increase in the fast time constant, the markedly slower decay in the EPSPs in *α*5*β*4‐KO neurons was determined primarily by the significantly larger amplitude for the slow component (Fig. 6B3).

## Discussion

Neurotransmission in both sympathetic and parasympathetic ganglia is mediated by ACh acting on nAChRs. In the SCG, our group and others found that *α*3*β*4, *α*3*β*4*α*5, and *α*3*β*4*β*2 receptors account for 55%, 24%, and 21%, respectively, of hetero‐pentameric nAChRs in the mouse SCG (Mao et al. 2006; David et al. 2010). Moreover, we previously found that loss of both the *β*2 and *α*5 subunits results in a pure population of *α*3*β*4 receptors in the SCG, whereas deleting either *β*4 alone or both the *α*5 and *β*4 subunits results in a pure population of *α*3*β*2 receptors (David et al. 2010).

Based on these findings, the *α*3 subunit is believed to be indispensable, and synaptic transmission in the SCG is believed to require the presence of *β*4 and/or *β*2 subunits. Consistent with this notion, SCG neurons isolated from mice lacking both the *β*2 and *β*4 subunits do not produce ACh‐activated whole‐cell currents (Xu et al. 1999b). Moreover, the putative critical role of the *α*3 subunit has been confirmed in the mouse SCG in a series of elegant experiments by Cooper and colleagues (Rassadi et al. 2005). Krishnaswamy and Cooper also reported that *α*3‐KO mice lack high‐affinity choline transporters at presynaptic terminals, suggesting that an activity‐dependent retrograde signal is required for maintaining presynaptic choline transport (Krishnaswamy and Cooper 2009).

### Why is CAP amplitude not affected in *β*4‐KO mice?

Xu et al. (1999b) previously reported major deficits in the autonomic nervous system of *β*4‐KO mice; in contrast, our data indicate that loss of this subunit has only a subtle effect on transmission in the SCG. Specifically, despite an 85% reduction in nAChRs in the SCG of *β*4‐KO mice, we found no difference between WT, *α*5*β*2‐KO, and *α*5*β*4‐KO SCG with respect to CAP amplitude. Given that CAP amplitude reflects the number of activated postsynaptic nerves, this finding suggests that despite the significant reduction of nAChRs in the *α*5*β*4‐KO SCG, synaptic receptors still activate the same number of postsynaptic neurons as in the WT SCG. We envision three possible explanations for this observation. First, postsynaptic sites in the SCG may contain considerably more nAChRs than are required for inducing an AP. Second, the relatively few receptors remaining in the *β*4‐KO ganglion may be sufficiently concentrated at the synapse in order to overcome the 85% reduction in overall receptor number. Finally, the number of receptors measured using IP may not provide an accurate measure of the actual number of receptors present in the membrane under normal physiological conditions.

With respect to the first possibility, this might be the case if a nonsaturating concentration of transmitter—and if the receptor's intrinsic properties—determines the magnitude of the postsynaptic current (Walmsley et al. 1998). In other words, with different numbers of postsynaptic receptors, a saturating concentration of transmitter would induce EPSPs of different amplitudes. Our finding that EPSP amplitude did not differ significantly between WT and *α*5*β*4‐KO ganglia is consistent with this mechanism. On the other hand, our modeling approach based on the effects of the nAChR antagonist HM supports the second and/or third possibilities listed above. Thus, both the nonsaturating concentration hypothesis and a higher number of cell‐surface receptors than predicted by IP could explain why CAP amplitude was similar between WT and *β*4‐KO ganglia.

Nevertheless, although CAP amplitude was not affected by deleting the *β*4 subunit, we found that deleting *β*4 significantly increased the threshold for generating CAPs. Given that the properties of the afferent nerve (for example, thickness of the myelin sheath) determine the stimulation threshold (Chen and Sandkuhler 2000; Hayami et al. 2010), we propose that in addition to reducing the overall number of nAChRs in the SCG, deleting the *β*4 subunit may also affect the properties of the cervical sympathetic trunk.

### Synaptic transmission is more susceptible to increasing stimulation frequency in *α*5*β*2‐KO and *α*5*β*4‐KO ganglia than in WT ganglia

At relatively low frequencies (i.e., ≤1 Hz), repeatedly stimulating the afferent nerve has little effect on EPSP amplitude. However, with increasingly higher frequencies, EPSP amplitude passes the following three stages: (1) initial facilitation (or depression) (Birks and Isacoff 1988), which is followed by (2) depression due to the depletion of presynaptic ACh stores (Bennett and McLachlan 1972a), followed by (3) steady‐state activity maintained by the synthesis of new ACh molecules (Bennett and McLachlan 1972b). In addition, synaptic transmission can also be affected by receptor desensitization (Papke et al. 2011) and/or by an extracellular buildup of K^+^ or decrease in extracellular Ca^2+^ (Galvan et al. 1979).

We measured synaptic transmission by recording CAP amplitude in response to increasing stimulation rate (up to 40 Hz) and found significant differences between the three genotypes. Specifically, high‐frequency stimulation had the largest effect on CAP amplitude in *α*5*β*4‐KO ganglia, followed by *α*5*β*2‐KO ganglia. Wang and colleagues previously reported that high‐frequency stimulation of the vagus nerve affects heart rate to a greater extent in WT mice than in *α*5‐KO and *β*4‐KO mice (Wang et al. 2002, 2003). Therefore, our findings support the view that receptor desensitization plays a critical role in the synaptic control of action potential‐mediated transmission (Papke et al. 2011), as do presynaptic mechanisms of transmitter release (Bennett and McLachlan 1972a,1972b).

Our unexpected finding that 5‐Hz stimulation causes paired‐pulse facilitation and increased CAP amplitude in WT ganglia—but not in *α*5*β*4‐KO or *α*5*β*2‐KO ganglia—are more difficult to explain. Given that an increase in CAP amplitude means that additional postganglionic neurons are recruited, it is reasonable to speculate that some postsynaptic neurons may have lost their presynaptic input during sample preparation. In WT mice, and specifically at a stimulation frequency of 5 Hz, these neurons could become activated by an overflow of ACh, whereas this mechanism might not play a role in *α*5*β*2‐KO or *α*5*β*4‐KO mice due to increased concomitant receptor desensitization occurring. Moreover, although retrograde signaling by postsynaptic receptor activation has been reported in the mouse SCG (Krishnaswamy and Cooper 2009), we currently have no evidence to suggest that this mechanism is affected in *α*5*β*2‐KO or *α*5*β*4‐KO mice.

The recruitment of additional postganglionic neurons—and thus the increased CAP amplitude in WT SCG ganglion—may also arise if a single presynaptic AP induces only subthreshold EPSPs in postganglionic neurons. This type of so‐called “weak input” could be particularly relevant in the *α*5*β*4‐KO SCG due to the dramatically reduced levels of nAChRs (David et al. 2010, but see above). However, under our experimental conditions, EPSP amplitude was similar between WT, *α*5*β*4‐KO, and *α*5*β*2‐KO ganglia. Furthermore, preganglionic stimuli delivered to neurons at their resting membrane potential always induced a suprathreshold EPSP, regardless of the genotype. On the other hand, the time constant of the EPSP decay in *α*5*β*4‐KO neurons differed significantly from both WT and *α*5*β*2‐KO neurons. Although the reason for this difference is unclear, the decay rate of cholinergic synaptic events may be affected by the burst duration of individual channels and/or repetitive ACh binding to receptors due to slowed ACh degradation (Katz and Miledi 1973; for review see Edmonds et al. 1995). However, the burst duration of *α*3*β*4 nAChRs was longer than *α*3*β*2 receptors when expressed in expressed in *Xenopus* oocytes (Nelson and Lindstrom 1999), and the potency of ACh at activating *α*3*β*2 receptors was similar to *α*3*β*4 and *α*3*β*4*α*5 receptors (Nelson et al. 2001; David et al. 2010).

### *α*3*β*4*α*5 receptors outnumber receptors that lack *α*5 subunits at the cell surface of WT SCG neurons

The nAChR blocker HM was previously shown to inhibit vagal stimulation–induced bradycardia more potently in mice that lack *α*5 subunits than in WT mice (Wang et al. 2002); in contrast, no such effect was observed in mice that lack *β*2 subunits (Wang et al. 2005). We can confirm the lack of effect of the *β*2 subunit (at least in this respect) by showing that the increased potency of HM at inhibiting CAP amplitude was similar between *α*5‐KO and *α*5*β*2‐KO ganglia.

Using a modeling‐based approach, we capitalized on the right‐shift in the HM concentration‐response curve in the presence of *α*5‐containing receptors in order to estimate both the IC_50_ of HM for a pure population of *α*3*β*4*α*5 receptors and the relative percentage of functional *α*3*β*4*α*5 receptors in WT ganglia. Our model revealed that HM inhibits *α*3*β*4*α*5 receptors with an IC_50_ of 568.6 *μ*M and that these receptors comprise 72% of nAChRs in the WT SCG; this latter finding is strikingly different from our data obtained using IP, in which only 24% of the nAChRs in WT SCG neurons were *α*3*β*4*α*5 (David et al. 2010).

Our results obtained with cultured SCG neurons support our ex vivo physiological data obtained using intact ganglia. Specifically, our modeling approach with culture neurons revealed that HM inhibits *α*3*β*4*α*5 receptors with an IC_50_ of 72.02 *μ*mol/L and that these receptors comprise 63% of functional nAChRs expressed in WT neurons. Based on both our ex vivo and in vitro data, we conclude that *α*3*β*4*α*5 receptors in WT SCG neurons greatly outnumber *α*3*β*4 receptors both at synaptic sites and across the entire cell surface.

Contrary to our observations, HM was slightly more potent in inhibiting currents in response the ACh in *Xenopus* oocytes expressing *α*5‐containing receptors (IC_50_: 3.5 *μ*mol/L), compared to oocytes expressing just *α*3*β*4 (IC_50_: 15.6 *μ*mol/L) (Papke et al. 2010). For the expression of receptors, Papke et al. (2010) injected *α*3:*β*4 and *α*3:*β*4:*α*5 RNA in a 1:1 or 1:1:1 ratio, respectively. However, the stoichiometry and the frequency of subtypes of the resulting receptors are unclear and may differ from receptors in the SCG.

Our conclusions are also at odds with a recent report showing that (*α*3)_2_(*β*4)_3_, but not (*α*3)_3_(*β*4)_2_ or (*α*3)_2_(*β*4)_2_ *α*5 receptors, are efficiently expressed in the plasma membrane of transiently transfected normal rat kidney cells (Crespi et al. 2018b). Whereas (*α*3)_3_(*β*4)_2_ receptors are retained in the endoplasmic reticulum because of the missing third *β*4 subunit carrying a LXM export motif, this motif is present in the *α*5 subunit. Still, (*α*3)_2_(*β*4)_2_ *α*5 receptors seem to be retained by a second limiting step at the level of the Golgi, thus preventing transport to the plasma membrane. We find, on the contrary, that (*α*3)_2_(*β*4)_2_ *α*5 clearly outnumber *α*3*β*4 receptors on the cell surface of SCG neurons and thus reason that experimental conditions with heterologously expressed receptors may not properly match receptor assembly, trafficking, targeting, and/or turnover of nAChRs in SCG neurons. As recently reviewed, the efficiency of assembly and trafficking varies widely depending on the nAChR subtypes and the cell type in which they are expressed (Crespi et al. 2018a).

The *α*5 nAChR subunit is expressed at much lower levels in the medial habenula (2.5% in rats, 6% in mice) than in the SCG, where it also co‐assembles with *α*3 and *β*4 (Scholze et al. 2012); moreover, this brain structure is critically important in nicotine dependence (Fowler et al. 2011; Frahm et al. 2011). Based on our observations in the SCG, it is reasonable to speculate that *α*3*β*4*α*5 receptors are likely expressed at the cell surface of medial habenula neurons at considerably higher levels than predicted from IP experiments (Grady et al. 2009; Scholze et al. 2012), which may explain the robust effect that the *α*5 subunit has on drug‐seeking behavior (Fowler et al. 2011; Frahm et al. 2011).

## Conclusions

By measuring synaptic transmission in isolated mouse SCG, we found significant differences between *α*5*β*4‐KO, *α*5*β*2‐KO, and WT mice with respect to inducing CAPs at increasing stimulation frequency and when exposing the ganglia to nicotine. Interestingly, however, the EPSP amplitude recorded in SCG neurons was similar between all three genotypes. By comparing the effects of HM between *α*5*β*2‐KO and WT ganglia and cultured SCG neurons, and by modeling the IC_50_ of a pure population of *α*3*β*4*α*5 receptors, we found that *α*3*β*4*α*5 receptors contribute to approximately 70% of CAP amplitude and ACh‐induced currents in the WT SCG. Thus, these physiological data provide compelling evidence that *α*3*β*4*α*5 receptors clearly outnumber *α*3*β*4 receptors at both synaptic sites and the cell surface of SCG neurons.

## Conflict of Interest

None declared.
